# College Notes

**Published:** 1893-04

**Authors:** 


### College Notes.

TERM examinations at the Buffalo medical colleges begin the latter part of the month.

THE medical department of Niagara University will hold its commencement Thursday, May 4, 1893. The programme outlined will consist of the meeting of the Alumni Association in the college building at ten A.M., commencement exercises at the Star Theater in the evening at eight P. M. The Rev. Mr. Slicer will deliver the address to the graduates, and Dr. Thomas Lothrop will deliver the valedictory address. This year closes the first decennial of this school. Physicians are cordially invited to attend these exercises.

THE commencement exercises of the University of Buffalo will be held at Music Hall, Tuesday evening, May 2, 1893. Diplomas will be granted to the graduates in the departments of medicine, pharmacy, and dentistry. The address of the evening will be delivered by Dr. Paul F. Mundé, of New York.

The Alumni Association of the medical department will meet in the morning, in Alumni Hall, for a business session, and in the afternoon scientific papers will be presented by Drs. C. C. Frederick, E. Clark, and others.

